# *In silico *comparison of transcript abundances during *Arabidopsis thaliana *and *Glycine max *resistance to *Fusarium virguliforme*

**DOI:** 10.1186/1471-2164-9-S2-S6

**Published:** 2008-09-16

**Authors:** Jiazheng Yuan, Mengxia Zhu, David A Lightfoot, M Javed Iqbal, Jack Y Yang, Khalid Meksem

**Affiliations:** 1 Department of Plant, Soil Sciences and Agriculture System, Southern Illinois University at Carbondale, Carbondale, IL 62901, USA; 2 Department of Plant Biology, Southern Illinois University at Carbondale, IL 62901, USA; 3 Department of Computer Science, Southern Illinois University at Carbondale, IL 62901, USA; 4 Harvard Medical School, Harvard University, Cambridge, MA 02140, USA; 5 Plants and Microbes Genomics and Genetics lab, Department of Plant, Soil Sciences, and Agriculture System, Southern Illinois University at Carbondale, Carbondale, IL 62901, USA

## Abstract

**Background:**

Sudden death syndrome (SDS) of soybean (*Glycine max *L. Merr.) is an economically important disease, caused by the semi-biotrophic fungus *Fusarium solani *f. sp. *glycines*, recently renamed *Fusarium virguliforme *(Fv). Due to the complexity and length of the soybean-Fusarium interaction, the molecular mechanisms underlying plant resistance and susceptibility to the pathogen are not fully understood. *F. virguliforme *has a very wide host range for the ability to cause root rot and a very narrow host range for the ability to cause a leaf scorch. *Arabidopsis thaliana *is a host for many types of phytopathogens including bacteria, fungi, viruses and nematodes. Deciphering the variations among transcript abundances (TAs) of functional orthologous genes of soybean and *A. thaliana *involved in the interaction will provide insights into plant resistance to *F. viguliforme*.

**Results:**

In this study, we reported the analyses of microarrays measuring TA in whole plants after *A. thaliana *cv 'Columbia' was challenged with fungal pathogen *F. virguliforme*. Infection caused significant variations in TAs. The total number of increased transcripts was nearly four times more than that of decreased transcripts in abundance. A putative resistance pathway involved in responding to the pathogen infection in *A. thaliana *was identified and compared to that reported in soybean.

**Conclusion:**

Microarray experiments allow the interrogation of tens of thousands of transcripts simultaneously and thus, the identification of plant pathways is likely to be involved in plant resistance to Fusarial pathogens. Dissection of the set functional orthologous genes between soybean and *A. thaliana *enabled a broad view of the functional relationships and molecular interactions among plant genes involved in *F. virguliforme *resistance.

## Background

Transcriptional changes play a major role in many plant defense processes [1]. Investigation of alterations in transcript abundance in functional genomics has provided unique opportunities to delve into gene functions by the comparison of species, tissue and time specific transcript accumulation for thousands of genes simultaneously [2-4]. The transcript abundances of the annotated genes of Arabidopsis, soybean and many other crops can be evaluated in parallel using high-density microarrays of sequenced cDNAs (AGI, 2000) or oligomers [5]. Microarray experiments have enabled the detection of significant variation in mRNA abundance and improved the understanding of the molecular mechanism of partial defense responses [6-9]. The host-pathogen interaction involved in incomplete, quantitative and partial resistance of soybean roots to *F. virguliforme *has been intensively investigated [9-11]. Transcription factors, chromatin remodeling proteins and transcript stabilizing factors are likely candidates to be involved. Regulated pathways are expected to include the synthesis of phytoalexins, signal molecules, cell wall deposition and carbon (C) and nitrogen partitioning.

Several studies suggested that disease resistance genes shared the same specificity across distantly related plant species [12-15]. The specificity of response was maintained, perhaps because of balancing selection, in lineages leading to multiple plant species [16]. However, it is difficult to conclude that a unified model of host-pathogen interactions has been determined because many of the genes underlying pathogen recognition were functional orthologs rather than the closest sequence homologous in different species.

Phytoalexins, phytoanticipins and signal molecules are three major natural products involved in plant defense with common precursors [17]. Phenylalanine ammonia-lyase (PAL; EC 4.3.1.5) expression has been associated with resistance to fungal pathogens in many plant species [18,19]. PAL catalyzes the deamination of phenylalanine to produce trans-cinnamic acid, the first step in the phenylpropanoid pathway leading to phytoalexins, lignins or coumarins. Multiple isoforms of the *pal *gene were identified in plants [20]. Manipulation of PAL, the first enzyme of the phenylpropanoid pathway together with the downstream enzymes such as cinnamate 4-hydroxylase (C4H; EC 1.14.13.11), diphenol oxidase (laccase; EC 1.10.3.2) and 4-hydroxycinnamoyl CoA ligase (4CL; EC 6.2.1.12), revealed an association with resistance to viral and fungal infection [21-23]. Reduction of phenylpropanoid biosynthesis in tobacco via down-regulation of PAL reduced local and systemic acquired resistance to fungal or viral infection [24,25]. Phenylpropanoid derived polymers like lignin also play an important role as a physical barrier against pathogen invasion [26]. Lignin, a complex racemic aromatic heteropolymer is the second most abundant cell wall polymer (after cellulose) and provides rigidity for the cell wall and a physical barrier against pathogens [27]. Lignin is synthesized from the phenylpropanoid metabolism reactions. These series of hydroxylation and O-methylation and conversion of side-chain carboxyl to an alcohol result in the building blocks of lignin, which is initiated by deamination of phenylalanine by the enzyme PAL where hydroxycinnamic acid esters play a central role [28,29]. 4CL is responsible for the CoA esterification of p-coumaric acid. Down-regulation of isozymes of 4CLs may alter metabolite concentrations other than those involved in lignin production, with a secondary effect on growth as a consequence [30]. Laccase was the first enzyme demonstrated to be able to perform lignin polymerization *in vitro*. Over-expressed laccase in the roots caused cell wall lignin deposition increases in the developing xylem [31,32,23]. Therefore lignin might be involved in the disease resistance mechanism of plant cells where wall fortification occurred in response to many pathogens and would be especially useful against root rots.

In plant, SnRKs possess a catalytic domain similar to that of sucrose non-fermenting-1 (SNF1) of yeast (*Saccharomyces cerevisiae*) and AMP-activated protein kinase (AMPK) of animals. SnRK1, SnRK2 and SnRK3 are the key members of SnRKs family but the SnRK1 subfamily appears to share direct structural and functional homology with the SNF1/AMPK family. The SnRK1 protein complex can be further divided into 3 subunits: the α subunit which is SNF1-like protein; the β subunit that is composed of SIP1-, SIP2- and GAL83-like proteins; and the γ subunit that is SNF4-like protein based on sequence structure and expression patterns [33,34]. Homologues of SnRKs occur in all kingdoms and they appear to be highly conserved among yeasts, animals and plants suggesting that they may play very similar roles across species [35]. SNF1 modulates the phosphorylation state of a number of metabolic enzymes whilst SnRK1 regulates several enzymes involved in sugar metabolism and cell energy metabolism [36,33]. In yeast, the function of SNF1 is to coordinate about 600 genes to respond to lower cellular glucose concentrations. Hong et al. [33] identified GAL83 as mediating carbon partitioning during the plant response to herbivore *Manduca sexta *attack. GAL83, a β-subunit of a heterotrimeric SnRK1, showed a decrease in source leaves whereas the abundance of the catalytic α-subunit of SNF remained unaltered. The herbivore-induced changes in sink-source relations in *Nicotiana attenuata *was regulated by the β-subunit of SnRK1 (SNF1-related kinase) protein kinase, GAL83. GAL83 silenced plants were unable to enhance root reserves, delay senescence or prolonged flowering following herbivore attack during early stages of development. In turn, SnRK1 can be used to alter resource allocation thereby plants may be equipped to better tolerate the pest attack [37].

**Figure 1:**
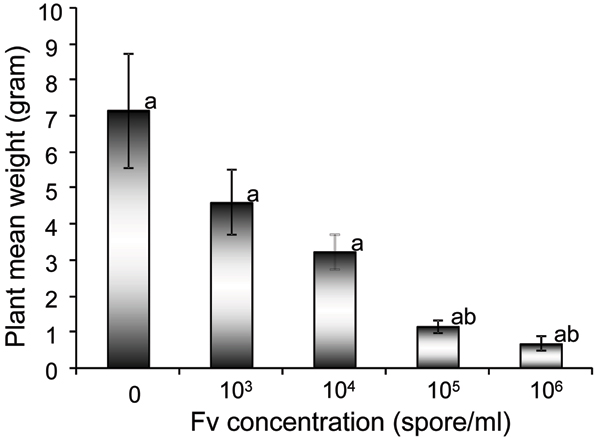
Arabidopsis thaliana responded to *F. virguliforme *with respect to the spore concentration. The plant exhibited a remarkable response to the concentration of the spore on the plant fresh weight (mean weight). The mean weights labeled with the same letter were not significantly different (*P *< 0.05).

Partial resistance can often be subcategorized as rate reducing resistance for fungal infections of roots [38,39]. Partial resistance may result from a reduced infection frequency, an extended latent period of infection, a reduced sporulation of the pathogen, or a combination of these [40-42]. However, the defense pathways that are induced during partial resistance do not share the same temporal and spatial patterns of gene expression observed in complete resistance [9,10,40,42,43]. Sudden death syndrome (SDS) of soybean caused by *F. virguliforme *[44] results from two distinct interactions. SDS has the root infection component, where the fungus exogenously penetrates root cell walls and infect specific cells and causes root rot whilst leaf scorch component, where some of the toxins produced in the roots are translocated to leaves and cause the leaf scorch. The leaf symptoms only occur in soybean [45,46] but the root rot occurs in all legumes, most dicots and some cereals. Both root rot and leaf scorch contributes to yield losses [38,47]. *Arabidopsis thaliana *is a host for many types of phytopathogens. We have observed that Arabidopsis is also an excellent model plant for *F. virguliforme *resistance (authors unpublished data). Arabidopsis was a host for *F. virguliforme *and the responses to the pathogen with nicely respect to the spore concentration (Figure 1). Our date showed that this ecotype demonstrated a rate reduction resistance to the fungal pathogen. Thus, *A. thaliana *should be useful for studying the interactions between plant and *F. virguliforme*.

Iqbal et al. [9] measured changes in TAs of 192 known plant defense and biotic/abiotic stress related genes in soybean roots at five time points over a period of 10 days after *F. virguliforme *inoculation. The cDNA arrays used were chosen from a soybean root cDNA library [48] and a subtractive hybridization experiment [9]. The temporal and spatial response differed among soybean genotypes with different numbers of SDS resistance genes. The responses were different in the partial resistance and susceptible genotypes among genes involved in the plant defense, signal recognition and transduction and metabolic processes. For most of the responding genes and all genotypes, there was an initial decrease in TAs in the inoculated roots. However, by later stages of post inoculation, the inoculated roots of the partially susceptible cultivars failed to increase abundance of any transcripts of known defense-related genes. In the most resistant cultivar, a set of 35 genes maintained at least a two-fold higher abundance at all time points. The increase in TA in RIL23 was in contrast to that observed in Essex, susceptible parent, where most of the ESTs showed either no change or a decreased TA [9].

Here, we analyzed the changes that occurred in the abundance of transcripts corresponding to 10,560 *A. thaliana *expressed sequence tags (ESTs) after *A. thaliana *cv 'Columbia' was treated with *F. virguliforme*. Reverse labeled slides were used. An ortholog analysis was exploited to understand the evolutionary roles of the regulated genes based on investigation of orthologous relationship between soybean and Arabidopsis. A set of resistance pathways involved in response to the pathogen infection in *A. thaliana *has been proposed. The comparison of the transcriptional activity in the resistance pathways between soybean and Arabidopsis after *F. virguliforme *pathogenesis allows for the examination of evolution of the disease response in both species.

## Results

### Transcript profile of Arabidopsis genes in response to *F. virguliforme *infection

Analysis of the microarray data demonstrated a significant variation within and between the slides after local and global normalization [49] and 6,109 genes correlated in both reverse labeled slides from the 10,560 EST arrayed. The position and label variations between two replications did not significantly alter the topography between slides. The fungal infestation caused 168 transcripts to increase in abundance more than 1 fold (>1 on Log2 scale). About 24 of the transcripts were increased in abundance more than 2 fold (Log2 scale). In contrast, only forty-two transcripts were observed to decrease in abundance by more than 1 fold (Log2 scale) and 14 of them went down more than 2 fold (Log2 scale) following *F. virguliform *infection. In addition, the number of the up-regulated genes was nearly four times more than that of down-regulated genes. Therefore, *A. thaliana *cv Columbia responses to *F. virguliforme *were more similar to resistant than susceptible soybean cultivars [9].

After the Arabidopsis EST-based microarray data were converted into Affymetrix 22 K array annotation, the 6,109 genes on the arrays were subjected to pathway construction using the MapMan platform [50]. The majority (3,541) of genes altered by the treatment (*P *< 0.05) could not be assigned to any of the known function (Table 1). In contrast, 2,568 genes were assembled into 14 major bins, each bin representing a set of related pathways (Table 1). Classification of the transcript abundances changed by *F. virguliforme *infestation showed several interesting features. There were 571 genes distributed among five protein metabolism related bins: synthesis, activation, posttranslational modification, degradation and folding. Bin16, secondary metabolism, bin29, protein metabolism and bin30, signal transduction were subjected further investigation. A large proportion of these protein metabolism related genes was assigned to bin29.2 (synthesis) and bin29.4 (posttranslational modification). Bin29.4 (protein post-translational modification) contained many genes involved in published plant defense schema [51]. The 186 genes encompassed in this bin category would allow various stages of plant response to pathogen challenge to be investigated. Of the 186 genes involved in protein posttranslational modification there were only 9 genes with significantly altered TAs. The synthesis of new proteins and the alteration of the activities of existing proteins by modification have been frequently reported to be important to plant pathogen resistance.

**Table 1:** Description of bin distributions in the MapMan platform.

**Bin**	**Name**	**Elements**	**p-value**
1.1	PS.lightreaction	59	0.04
1.1.4	PS.lightreaction.ATP synthase	6	0.01
	TCA/org. transformation.other organic acid		
8.2.11	transformaitons.atp-citrate lyase	2	0.03
10.2	cell wall.cellulose synthesis	14	0.01
	lipid metabolism.FA synthesis and FA elongation.long		
11.1.9	chain fatty acid CoA ligase	5	0.004
12	N-metabolism	13	0.03
12.2	N-metabolism.ammonia metabolism	8	0.04
	amino acid metabolism.synthesis.aspartate		
13.1.3.4	family.methionine	9	0.046
16.1.4	secondary metabolism.isoprenoids.carotenoids	6	0.04
	secondary metabolism.phenylpropanoids.lignin		
16.2.1	biosynthesis	18	0.006
16.7	secondary metabolism.wax	4	0.027
16.8.2	secondary metabolism.flavonoids.chalcones	3	0.006
	hormone metabolism.brassinosteroid.synthesis-		
17.3.1.1.1	degradation.BRs.DET2	3	0.02
19.1	tetrapyrrole synthesis.magnesium chelatase	2	0.03
26.3	misc.gluco-, galacto- and mannosidases	22	0.01
27.1	RNA.processing	71	0.01
	RNA.regulation of transcription.TCP transcription factor		
27.3.29	family	6	0.04
29.1	protein.aa activation	32	0.02
29.2	protein.synthesis	200	0.004
	protein.synthesis.chloroplast/mito – plastid ribosomal		
29.2.1	protein	36	0.007
	protein.synthesis.chloroplast/mito – plastid ribosomal		
29.2.1.1	protein.plastid	21	0.04
29.2.2	protein.synthesis.misc ribososomal protein	106	0.01
29.4	protein.postranslational modification	186	0.03
	protein.postranslational modification.kinase.receptor like		
29.4.1.51	cytoplasmatic kinase I	2	0.03
29.5.11.4.3.2	protein.degradation.ubiquitin.E3.SCF.FBOX	58	0.01
29.6	protein.folding	25	0.006
30	signalling	267	0.02
30.3	signalling.calcium	59	0.01
35	not assigned	2150	0.02
35.2	not assigned.unknown	1191	0.003

The bin number of the microarray was also denoted from 1–100 and it was only shown for the bins with significant changes in transcript abundance at *P *< 0.05.

Further *in silico *analysis, the platform of the Arabidopsis Interactions Viewer [52] was used to investigate the potential molecular protein-protein interaction based on the observed transcript changes. When the 186 genes were deployed in the Arabidopsis Interactions Viewer, there were more than 745 interlogs obtained. However, only two hubs (AMP kinase and phosphatase associated protein 46 and TAP46) were identified if these 9 genes were loaded into the Arabidopsis Interactions Viewer under higher stringency.

### Secondary metabolism and lignin biosynthesis pathways

**Figure 2:**
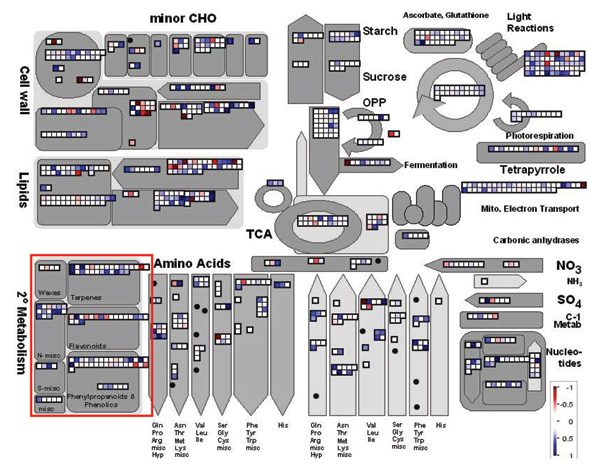
TA changes in responses of Arabidopsis plant to *F. virguliforme*. The secondary metabolism in the MapMan program [50] was marked in red square. Blue and Red squares were denoted increased (positive) and decreased (negative) transcript abundances (TAs), respectively. The bar was shown on log2 scale.

The 31 genes which had been classified as potentially being involved in secondary metabolism (bin 16; Table 2; Figure 2) were prominent. Several other genes that were reported to be involved in plant responses to pathogen attack were found within the schema. They were subdivided into pathways leading to isoprenoid, phenylpropanoid and lignin biosynthesis. The 18 genes were mapped into bin 16.1.4 (isoprenoids, carotenoids) included genes involved in signaling, signaling-calcium, cell wall and cellular synthesis and 13 genes were mapped into bin16.2.1 (phenylpropanoids) and bin16.7 (waxes).

**Table 2:** The protein-protein interaction carried out by the Arabidopsis Interaction Viewer on Bin 16.

**Locus**	**Description**	**Fold change**
**At3g10340**	**phenylalanine ammonia-lyase**	**3.395**
**At2g23910**	**cinnamoyl-CoA reductase**	**2.688**
At4g09500	glycosyltransferase family protein	1.131
At5g12210	geranylgeranyl transferase type II beta subunit	1.117
At4g17190	farnesyl pyrophosphate synthetase 2	1.032
At5g62790	1-deoxy-D-xylulose 5-phosphate reductoisomerase	1.013
At2g40230	transferase family protein	0.924
At1g74020	atss-2 strictosidine synthase	0.899
At1g26410	FAD-binding domain-containing protein	0.889
At3g10230	lycopnene beta-cyclase	0.887
At1g62570	flavin-containing monooxygenase family protein	0.826
At5g57840	transferase family protein	0.796
At1g08550	violaxanthin de-epoxidase precursor	0.665
At4g34540	isoflavone reductase family protein	0.665
At1g35190	oxidoreductase	0.633
At2g29330	tropinone reductase, putative	0.603
At4g16330	oxidoreductase	0.559
At1g06570	1;4-hydroxyphenylpyruvate dioxygenase	0.552
At1g58180	carbonic anhydrase family protein	0.541
At4g33360	terpene cyclase	0.523
**At3g21240**	**4-coumaroyl-CoA synthase 2**	**-0.057**
**At2g30490**	**cinnamic acid 4-hydroxylase**	**-0.274**
**At5g13930**	**chalcone synthase**	**-0.479**
At3g55120	chalcone-flavanone isomerase	-0.51
At3g51240	naringenin 3-dioxygenase/flavanone 3-hydroxylase	-0.52
**At4g34230**	**cinnamyl-alcohol dehydrogenase**	**-0.556**
At4g39330	mannitol dehydrogenase	-0.645
At1g17050	geranyl diphosphate synthase	-0.821

Key genes were in bold. The value was on Log 2 scale.

**Figure 3:**
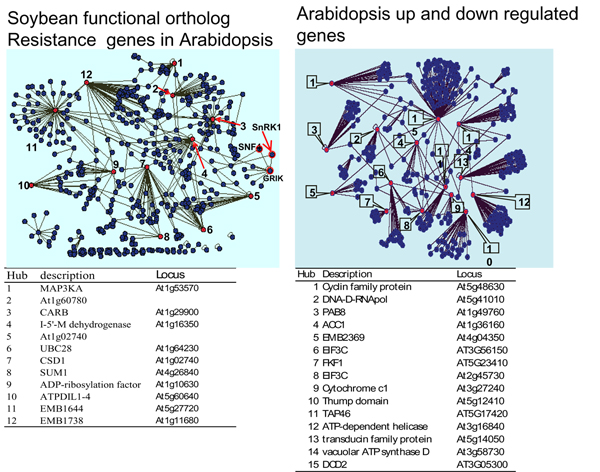
Network of protein-protein interaction of *A. thaliana *and soybean infested with *F. virguliforme*. The networks were generated by Cytoscape [67] and visual displays were saved as Cytoscape graphs. Interaction hubs were marked by red.

The transcriptional analysis of genes from the multiple branches of the phenylpropanoid pathway showed that 6 of the 31 genes were significantly altered in TA (Table 2). Further, TA changes inferred that synthesis of cinnamic acid in Arabidopsis was one of the early responses to *F. viguliforme *infection. Phytoalexins and phytoanticipins serve both structural and metabolic functions in disease resistance. Our microarray data showed that the resistance response of the phenylpropanoid pathway was different in Arabidopsis and soybean [9]. The microarray data showed that the phenylpropanoid pathway was activated during the resistance response in the Arabidopsis. In contrast to soybean [9], despite the fact that the PAL was induced in this metabolic pathway, other major genes such as cinnamic acid 4-hydroxylase (C4L), chalcone synthase (CHS) and cinnamyl-alcohol dehydrogenase (CAD) in the phenylpropanoid pathway were all suppressed (Table 2), indicating that soybean and Arabidopsis did not share similar strategies in the specific pathway to resistance *F. virguliforme*. Based on the up-regulation of ESTs, total six genes in the phenylpropanoid pathway had more than 1-fold increase in abundances (Log2 scale). However, the rest of genes involved in major branches in the pathway were either down-regulated or no significant increase in TAs (Table 2). The suppression of these branches of the phenylpropanoid pathway seemed somehow different from the soybean response to the pathogen [9]. Since transcripts of those enzymes leading to the synthesis of flavonols, terpenes and proanthocyanidins, decrease in abundances of down-stream genes in the procedure of the resistance response in the pathway but increase in PAL and cinnamoyl-CoA reductase transcripts during this same time suggesting the existence of a potential bypass to synthesize secondary metabolites in the defense response. After the 192 soybean genes have been converted into 158 functional orthologs of Arabidopsis genes, comparison of the protein-protein interaction network in both Arabidopsis and soybean demonstrated a high specificity trend in gene regulation of the two species (Figure 3). There were 12 hubs identified in the 158 functional orthologous soybean genes whilst more than 15 network hubs were observed to mediate the resistance in the up-regulated Arabidopsis genes. Unfortunately, no resistance pathway was generated from our Arabidopsis microarray data by the Arabidopsis Interactions Viewer program in the bin16 and no significant interlog among genes in bin16 of the data obtained.

### SNF1 (sucrose non-fermenting-1)-related protein kinase (SnRK1, AKIN10) gene

**Figure 4:**
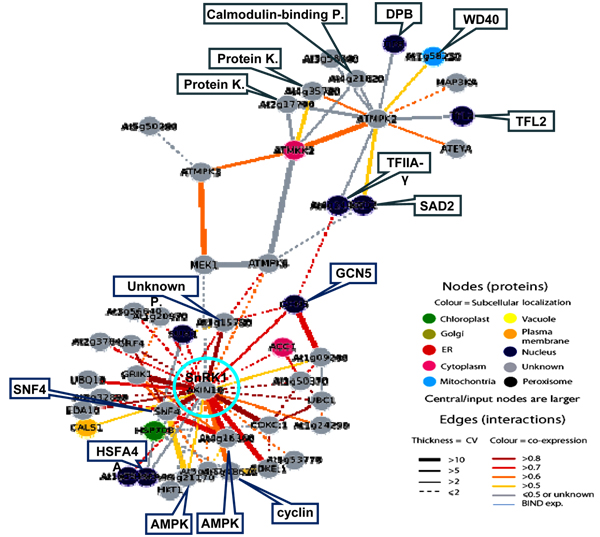
Interlogs of Bin30 based on Arabidopsis Interactions Viewer [52] SNF1 (sucrose non-fermenting-1)-related protein kinase (SnRK1) gene was positioned in lower part of the figure by the computer program.

**Figure 5:**
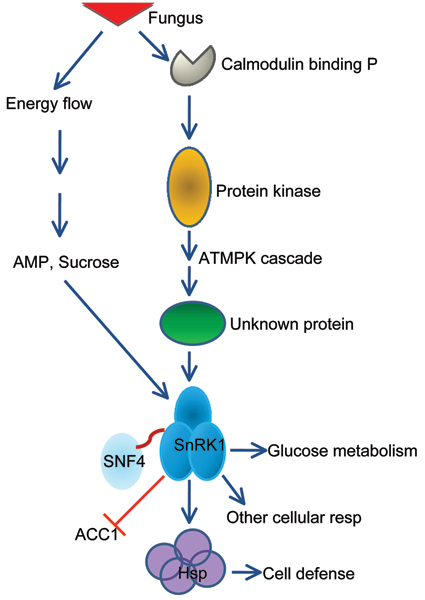
Simplified diagram of putative SnRK1 pathway. SnRK1 hub was marked by a green ring.

When all 50 genes altered in TA from in bin30 (signaling) were subjected to Arabidopsis Interactions Viewer platform, more than 300 interlogs were identified. The genes clustered into three major categories based on both interlog confidence value and Pearson correlation coefficient. The first group of the interlogs was mediated around MAP (mitogen-activated protein) kinases, the second group was centered on SNF1-related protein kinase 1 (SnRK1; Figure 4), and the third group was formed around ATMPK (Table 3). The second group contained 32 genes altered in TAs and this group was composed of three putative major nodes with a total 111 hits and 443 interlog confidence values (Table 3). A putative signal transduction pathway was derived from the interaction network (Figure 5). The results suggested that the SnRK1 gene could be important for coordinating the signal assembly of a cellular apparatus associated with the "endogenous fuel gauge" [53], since of the 31 genes in the reaction center may be differentially regulated by the endogenous AMP and sugar content in order to maintain cell defense. As shown in Figure 3, SnRK1 was centered among 31 interacting genes. The group was composed of 3 clades and most genes corresponded to the signal transduction cascade and cellular responses (data not shown).

**Table 3:** Overview of protein-protein interaction of bin30 based on Arabidopsis Interactions Viewer program.

**Protein 1**	**Protein 2**	**Interolog Confidence Value**	**Interolog Confidence**	**Pearson Correlation Coefficient**	**Protein2 Subcellular Localisation**	**Protein1 Annotation**	**Protein2 Annotation**	**Total hits**	**Protein2 fold change**
At3g45640	At4g26070	27	High	0.65	N/A	MAP kinase 3	MAP kinase kinase1	9	
At3g45640	At4g29810	9	Medium	0.633	cytosol	MAP kinase 3	MAP kinase kinase2	3	1.02
At3g45640	At5g50260	1	Low	-0.053	N/A	MAP kinase 3	cysteine proteinase	1	
At3g01090	At1g09020	287	High	0.822	N/A	SnRK1	Sucrose NonFermenting 4	41	
At3g01090	At3g45240	27	High	0.829	N/A	SnRK1	GRIK kinase 1	9	1.17
At3g01090	At5g48630	24	High	0.661	N/A	SnRK1	cyclin family protein	6	2.93
At3g01090	At4g16360	20	High	0.732	N/A	SnRK1	AMP-activated protein kinase	10	1.16
At3g01090	At5g10270	16	High	0.892	N/A	SnRK1	cyclin dependent kinase C 1	4	1.2
At3g01090	At5g63610	16	High	0.726	N/A	SnRK1	HUA ENHANCER 3	4	
At3g01090	At1g15780	6	Medium	0.878	N/A	SnRK1	unknown protein	3	1.16
At3g01090	At4g05320	6	Medium	0.74	N/A	SnRK1	polyubiquitin 10	3	1.12
At3g01090	At1g01960	4	Medium	0.829	N/A	SnRK1	G-nucleotide exchange factor	2	
At3g01090	At1g09200	4	Medium	0.565	N/A	SnRK1	histone H3	2	1.4
At3g01090	At1g24290	4	Medium	0.694	N/A	SnRK1	AAA-type ATPase protein	2	
At3g01090	At3g54610	4	Medium	0.766	nucleus	SnRK1	Histon acetyltransferase 1	2	
At3g01090	At4g18880	4	Medium	0.199	nucleus	SnRK1	HS transcription factor A4A	2	
At3g01090	At5g21170	3	Medium	0.571	N/A	SnRK1	AMP-activated protein kinase	3	2.17
At3g01090	At1g05570	2	Medium	0.586	PM	SnRK1	callose synthase	2	
At3g01090	At1g14400	1	Low	0.831	N/A	SnRK1	ubiquitin carrier protein 1	1	1.15
At3g01090	At1g16030	1	Low	-0.04	plastid	SnRK1	HS protein 70B	1	
At3g01090	At1g20970	1	Low	0.744	N/A	SnRK1	adhesin-related	1	
At3g01090	At1g35160	1	Low	0.807	NC, cytosol	SnRK1	general regulatory factor 4	1	
At3g01090	At1g36160	1	Low	0.747	PM, cytosol	SnRK1	acetyl-CoA carboxylase	1	0.33
At3g01090	At1g50370	1	Low	0.854	N/A	SnRK1	ser/thr protein phosphatase	1	
At3g01090	At1g75560	1	Low	0.741	nucleus	SnRK1	zinc knuckle family protein	1	
At3g01090	At2g32850	1	Low	0.837	N/A	SnRK1	protein kinase family protein	1	1.24
At3g01090	At2g37840	1	Low	0.71	N/A	SnRK1	protein kinase family protein	1	
At3g01090	At2g43790	1	Low	0.665	N/A	SnRK1	ATMPK6	1	0.86
At3g01090	At3g56640	1	Low	0.777	N/A	SnRK1	exocyst complex subunit	1	
At3g01090	At4g10310	1	Low	0.385	N/A	SnRK1	sodium ion transporter	1	
At3g01090	At4g26070	1	Low	0.297	N/A	SnRK1	MAP kinase kinase 1	1	
At3g01090	At4g26840	1	Low	0.72	nucleus	SnRK1	small ubiquitin-like modifier 1	1	0.79
At3g01090	At5g44740	1	Low	0.686	N/A	SnRK1	UMUC-like DNA repair protein	1	
At3g01090	At5g53770	1	Low	0.668	N/A	SnRK1	nucleotidyltransferase	1	
At1g59580	At4g29810	12	High	0.621	cytosol	MAP kinase 2	ATMKK2	12	1.02
At1g59580	At2g31660	8	Medium	0.521	nucleus	MAP kinase 2	SAD2	8	
At1g59580	At2g17700	6	Medium	0.358	N/A	MAP kinase 2	protein kinase	6	0.88
At1g59580	At1g58230	4	Medium	0.57	mitochondria	MAP kinase 2	WD-40 repeat protein	4	
At1g59580	At2g35320	4	Medium	0.601	N/A	MAP kinase 2	protein tyrosine phosphatase	4	
At1g59580	At4g21820	4	Medium	0.099	N/A	MAP kinase 2	calmodulin-binding protein	4	
At1g59580	At5g17690	4	Medium	0.447	nucleus	MAP kinase 2	terminal flower 2	4	
At1g59580	At3g58040	2	Medium	0.278	N/A	MAP kinase 2	seven in absentia protein	2	
At1g59580	At4g24440	2	Medium	0.345	nucleus	MAP kinase 2	TFIIA-gamma	2	
At1g59580	At4g35780	2	Medium	0.657	N/A	MAP kinase 2	protein kinase protein	2	
At1g59580	At5g03415	2	Medium	0.269	nucleus	MAP kinase 2	DPB__DPB	2	
At1g59580	At1g53570	1	Low	0.685	N/A	MAP kinase 2	MAP kinase kinase kinase 3	1	1.15
At1g59580	At1g59580	1	Low	1	N/A	MAP kinase 2	ATMPK2	1	1.47

Fold change was on Log2 scale.

Upstream of the SnRK1 signal cascade, the ATMPK cascade may be connected to cellular calcium (through calmodulin binding protein) and protein kinases (Figure 4, 5). In animals, LKB1 (also called STK11, a recently identified tumor suppressor gene) is required for the activation of AMPK in response to cellular concentrations of AMP induced by cellular stresses. The increase in AMP promotes phosphorylation by LKB1 [54]. The SnRK1 shared 33% identify with one of the mouse LKB1 paralogs (BAA76749) and there was a significant interlog predicted between the SnRK1 and LKB1. Pathogen elicitor or biotic and abiotic stresses may activate MAPK pathway through an unknown protein. On the other hand, cellular C and AMP concentrations may also trigger the AMPK to induce SnRK1via SNF4. However, the role of the AMPK of Arabidopsis and its molecular significance and molecular fragments located immediately in the upstream of SnRK1 are still unknown. The TAs of the AMPK (At5g21170) and cyclin family protein (At5g48630) were significantly increased by *F. virguliform *but ACC1 (acetyl-CoA carboxylase 1) was suppressed (Table 3). Analysis of functional orthologs of the soybean genes in Arabidopsis indicated that significant variations existed between two species facing the same pathogen. Interestingly, SnRK1 and SNF4 were also found in the soybean resistance network (Figure 3).

### Comparison of the fungal resistance genes between Arabidopsis and soybean

168 genes with altered TAs in the Arabidopsis microarray data shared homology to the 192 soybean genes involved in resistance, signal transduction, plant defense and transport of metabolites. Those TAs from both species were functionally clustered into three major groups (data not shown) using CLUSTAL X [55]. The results of multiple alignments were subjected to phylogenetic analysis with algorithm of the MEGA4 software [56] using the Maximum Parsimony analysis with Kimura two parameter distances. The reliabilities of each branch point were assessed by the analysis of 500 bootstrap replicates. The Maximum Parsimony (MP) analysis showed that these genes possessed homology by evolutionary descent. Maximum Parsimony phylogenetic analyses partitioned these resistance genes in Arabidopsis and soybean [9] jointly into 7 clades (denoted I-VII, data not shown). Each clade contained various members. Some clades could be further subdivided into subclasses. Clade III contained members that were the most divergent compared with those encoded by the other clades. Interestingly, there were several ESTs that were only found in one species. Majority families or subfamilies in the higher nodes tended to give low bootstrap values (data not shown).

## Discussion

It is well-known that over-expression PAL in tobacco plant can effectively be used against a virulent fungal pathogen *Cercospora nicotiana *infection and also altered the lesion phenotype inoculated with TMV [57]. Reduction of phenylpropanoid biosynthesis in tobacco via down-regulation of PAL reduces local and systemic acquired resistance to fungal or viral infection [27,28]. The major function of PAL is to catalyze phytoalexins and phytoanticipins production and thereby reduce disease severity [58]. Constitutive over-expression of the PAL gene from the tropical pasture legume *Stylosanthes humilis *in tobacco plants provided resistance to *Cercospora nicotianae *and to pathogen *Phytophthora parasitica *pv. *Nicotianae *[59]. The key enzymes of phenylpropanoid pathway, PAL, C4L and CHS were all induced in soybean resistance to *F. virguliforme *[9]. The global expression analysis of the Arabidopsis plant challenged by *F. virguliforme *appeared to be different. The fungal pathogen affected the secondary metabolism not only in the phenylpropanoid pathway but also via pathways leading to other cellular functions (terpenes, phenolics, special N compounds metabolism). Transcripts of the PAL gene encode the key enzyme feeding all these branches. Therefore, the decreases in most key transcripts at the times when PAL and cinnamoyl-CoA reductase transcripts increases in abundance is a novel finding showing that the synthesis of PAL and cinnamoyl-CoA reductase transcripts is with negative correlation to the activity of the other genes during the pathogen stress response. Perhaps in *A. thaliana*, the phenylpropanoid pathway did not directly participate in the cascade of reactions elicited during the defense and probably, the biosynthesis of phytoalexins and phytoanticipins was regulated in different fashions in soybean and Arabidopsis. Transcripts of genes involved in the synthesis of lignin, flavonols, anthocyanins and proanthocyanidins (data not shown) seemed to be down-regulated during the periods of time when the genes involved in the synthesis of cinnamic acid was up-regulated. Arabidopsis lacks isoflavonoid and phytoalexins and produces mainly camalexin rather than a cocktail of isoflavonoids [60]. Therefore, in Arabidopsis the flavonoids (anthocyanins, proanthocyanidins, flavones and flavonols) may indeed not play a significant role in defense. In contrast, there was evidence that cinnamoyl alcohol dehydrogenase (CAD) was induced rapidly in Arabidopsis infected with *Xanthomonas *[61] and CAD is in the pathway that leads to lignin biosynthesis. In the phenylpropanoid pathway, key genes like PAL are encoded by a group of genes showing a great degree of sequence diversity. Significantly up-regulated PAL transcripts support for the hypothesis that in Arabidopsis expression of the PAL is significantly associated with pathogen infection. However, the PAL over-expression plants caused a significant reduction in growth and delayed flowering. These phenotypes may also be related to energy status or amino acid pool availability being altered by PAL.

It has been shown that the addition of sugar activates genes related to disease resistance [62]. The metabolic change in the pathogenesis could negatively affect cellular C content based on the expression of sugar-repressive nuclear genes and may be involved in the regulation of these genes [63]. Therefore, there should be other mechanisms for maintaining the cellular C concentrations. Moreover, metabolic movement should provide some explanation. Our results infer that the metabolite profiles may affect the status of disease resistance in the Arabidopsis plant. The response to C stresses is probably the key role of SnRK1 in eukaryotes [64]. SnRK1 may represent a primordial protein that in plants plays an important role in all resistances. It should be noted that SnRK1 interacts with 31 proteins, and each is located up- and down-stream of the gene in the interactome. Perhaps, the SnRK1 sequence has shared motifs with the rest of other proteins in the network. Interestedly, the presence of proteins in Arabidopsis related to three subunits of the SnRK1 protein might indicate an early duplication of these subunits in the process of evolution. To date, it is still not clear how, during the course of evolution of multicellular organisms, the SnRK1 system acquired the ability to be a signal system. One important implication is that SnRK1 serves as one of the hubs for the signal cascades in the plant. Although the relative specificity of SnRK1 in plants is yet unknown, it is possible that further investigation of such role may allow us to draw new resistance pathways based on energy status. Indeed, plant SnRK1 associated with herbivore tolerance was recently identified [53]. Our results suggest that interaction between SnRK1 with its network partners may be induced by the fungal pathogen. Further studies of the gene and the interacting partners should help to uncover important specific aspects of SNF1-mediated signaling during pathogen infection.

Orthologous genes are defined by direct evolutionary descent and should play similar developmental or physiological roles. Several gene groups were identified according to the outcome of functional orthologous analysis in this study, showing same functional orthologous relationships in these two species. In our primary results, the 1 to 1 ratio of the orthologous relationship was found among those several paired groups, indicating these gene groups were descended from a common ancestor and corresponded to well-conserved functions. These 1 to 1 ortholog classes are presumed to represent conserved functions in Arabidopsis and soybean, but they shared diverse bootstrapping value. Diversification following gene duplication may have occurred but the degree is still unknown. Twenty-eight pairs of the genes from both species were taxoned together and each pair possessed unique bootstrapping value and Kimura phylogenetic distance (data not shown). Based on phylogenetic analysis, the organization of soybean phenylalanine ammonia-lyase (PAL) proteins were very similar to that of the Arabidopsis PAL proteins, implying that the soybean and Arabidopsis PAL proteins analyzed here were all derived from a common ancestor. The fact that these clades were formed by both Arabidopsis and soybean PAL genes gave a suggestion that these PAL genes existed before the divergence of monocots and dicots. Interestingly, fair numbers of EST sequences were not found in the other organism, even in closely related gene families that were associated with the fungal resistance. However, detailed screening phylogenetic relationship among those resistance genes is necessary for detection of the gene evolution.

Plants possess an ability of resistance to most potentially pathogenic microbes. Gene transcriptional changes are critical for many plant defense processes [1]. Soybean SDS consists of root infection and leaf scorch. With the limitations in the study of solely predicted gene interactions, definite conclusions about the nature of the resistant response to *F. virguliforme *infection cannot yet be made. Investigation on differentially expressed genes of Arabidopsis and soybean in response to *F. virguliforme *can lead to better understanding of the mechanisms of resistant crops for certain disease. Microarray experiment allows interrogation of tens of thousands of genes simultaneously. To understand the molecular interactions involved in *F. virguliforme *resistance, we have integrated results from the soybean SDS resistance with Arabidopsis DNA microarray studies by *in silico *analyses. The results of this study can be used as a model system to facilitate the understanding of plant resistance to *F. virguliforme*.

## Methods and materials

### Plant materials

*Arabidopsis thaliana *cv. Columbia plants were germinated from seeds under conditions of 16 h photoperiod (500 μE/M2/sec) with temperatures at 22°C day/18°C night and 80% (v/v) relative humidity in a growth chamber. Plants were grown on rafts floating on liquid MS medium [65]. There were 30 plants per treatment arranged in a randomized complete block with three plants per treatment per block. Twenty-one days after planting, synchronously growing plants were selected and collected.

### Inoculation of roots with *F. virguliforme *spores

The *F. virguliforme *isolate 'Mont-1' was obtained from Dr. Shiuxian Li at the National Soybean Research Laboratory (Urbana, IL). *F. virguliforme *was cultured on potato dextrose agar medium (PDA, Difco, Detroit, MI) supplemented with 80 mg ml-1 tetracycline and a few drops of Tween 20. A spore suspension of *F. virguliforme *isolate 'Mont-1' was prepared as described [66]. The spore suspension, at 5 × 10^4 ^spore ml-1 with sterile distilled water, was made by adding *F. virguliforme *spores from several *F. virguliforme *culture plates and was continuously stirred on a stir flask to keep a uniform suspension. The spore suspension was poured on the growth medium for the infested plants and the same volume of sterile distilled water was added to non-infested plants.

### RNA isolation and microarray procedure

RNA was isolated separately from both inoculated and non-inoculated roots of 30 *A. thaliana *plants that were bulked and ground to a fine powder in liquid nitrogen. RNA was extracted with a RNeasy Plant Mini Kit (Qiagen, Valencia, CA) according to the manufacturer's instructions. RNA samples were treated with DNase in order to remove any residual DNA using the RNase-free DNase kit (Qiagen GmbH, Hilden, Germany) according to the manufacturer's instructions. After DNase treatment, RNA was purified on RNeasy mini spin columns (Qiagen, Valencia, CA). The quantity and quality of the RNA recovered was determined by spectrophotometry at 260–280 nm and electrophoresis on a 1.2% (w/v) agarose, 20% (v/v) formaldehyde gel. The microarray hybridization and slide scanning were carried out by the facility at AFGC . Microarrays (16561.xls and 27314.xls at  were used in the experiment. The mRNA samples corresponding to treatment (infested) and control (non-infested) was labeled during the cDNA synthesis with Cy3- or Cy5- labeled dUTP and with one technical replicate labeled by reversed dye compared to the first hybridization.

### Data analysis

A visualization software, MapMan was used to perform a gene ontology where the set of Arabidopsis genes of the microarray was assigned based on the non-redundant and hierarchically categorized assignment of BINs and sub-BINs at TIGR (The Institute for Genomic Research). The ontology was derived from the Affymetrix 22 K array corresponding to similar or sub-modal biological functions [50]. The classification of Image Annotator in the software was also used to diagram the data display. The changes were expressed relative to those in pathogen challenged roots. Transcripts that increased in abundance were denoted in blue, and transcripts were decreased in abundance were denoted in red. In the scale used for the visualized data, a 1-fold change (Log2 scale) was required to produce a visible coloration, and the scale saturates at a 3-fold (log2 scale) change. Blue and Red squares were denoted increased (positive) and decreased (negative) transcript abundances (TAs), respectively. The bar was shown on log2 scale. The Arabidopsis Interaction Viewer queries a database of 19,979 predicted and 1,499 confirmed interacting proteins. The predicted interactions (interologs) were generated by Geisler-Lee et al. [52]. Output of two interlogs was transferred to the Cytoscape software environment [67] for network visualization and modeling against each other in order to catalog all of their conserved pathways and gene interaction networks. The program was equipped with a plug-in architecture for customizing applications. The visual displays were saved as a Cytoscape graphs.

Stringent quality control measures were applied to all stages of data analysis. The Microarray data were normalized by local (local background value was subtracted from the intensity value of each spot) and global metrics. The procedures described by Pevsner [49] were followed to adjust for differences in the intensity of the two labels. Coefficients of means and variances on the signal intensities in each channel and ratio of signals from two replicates were calculated by our C++ program (available on request), which was also used to handle the missing and extra data values. The average ratio for a signal microarray from two replicates was computed by the equation of [Ratio1st+ (1/Ratio2nd)]/2. The Student's t-test was used to determine the statistical significance for genes considered between and within Microarrays slides and the plant mean weight differences (P < 0.05).

### Phylogenetic analyses

Functional sequence analysis was performed on amino acid sequences using Clustal X [55] with the default settings. Nucleotide sequences were aligned with Clustal W [68]. The results of multiple alignments were subjected to phylogenetic analysis using the algorithm of the MEGA package version 4.0 using the Maximum Parsimony analysis with Kimura two parameter distances [56]. The Maximum-Parsimony was assessed by 500 bootstrap replicates. Only nucleotide sequences were employed in phylogenetic analysis based on higher stringy consideration for the phylogenetic tree construction.

## Competing interests

The authors declare that they have no competing interests.

## Authors' contributions

DAL and JI envisioned the research. JY and JI grew plants, infested them and made RNA. AFGC carried out the microarrays. JY and MZ did data analysis. All authors helped in manuscript preparation. All authors read and approved the final manuscript. DAL, KM and JI envisioned the research.  
